# Decentralized Care for Rifampin-Resistant Tuberculosis, Western Cape, South Africa

**DOI:** 10.3201/eid2703.203204

**Published:** 2021-03

**Authors:** Sarah V. Leavitt, Karen R. Jacobson, Elizabeth J. Ragan, Jacob Bor, Jennifer Hughes, Tara C. Bouton, Tania Dolby, Robin M. Warren, Helen E. Jenkins

**Affiliations:** Boston University, Boston, Massachusetts, USA (S.V. Leavitt, K.R. Jacobson, E.J. Ragan, J. Bor, T.C. Bouton, H.E. Jenkins);; Boston Medical Center, Boston (K.R. Jacobson, E.J. Ragan, T.C. Bouton);; University of the Witwatersrand, Johannesburg, South Africa (J. Bor);; Stellenbosch University, Stellenbosch, South Africa (J. Hughes, R.M. Warren);; Brown University, Providence, Rhode Island, USA (T.C. Bouton);; Green Point Tuberculosis Laboratory, Cape Town, South Africa (T. Dolby);; South African Medical Research Council Centre for Tuberculosis Research, Cape Town (R.M. Warren);; Department of Science and Technology–National Research Foundation Centre of Excellence for Biomedical Tuberculosis Research, Cape Town (R.M. Warren)

**Keywords:** treatment, hospitalization, drug resistance, routinely collected data, health policy, antimicrobial resistance, rifampin, tuberculosis, decentralized care, respiratory infections, bacteria, South Africa, rifampin-resistant

## Abstract

In 2011, South Africa implemented a policy to decentralize treatment for rifampin-resistant tuberculosis (TB) to reduce durations of hospitalization and enable local treatment. We assessed policy implementation in Western Cape Province, where services expanded from 6 specialized TB hospitals to 406 facilities, by analyzing National Health Laboratory Service data on TB during 2012–2015. We calculated the percentage of patients who visited a TB hospital <1 year after rifampin-resistant TB diagnosis, the median duration of their hospitalizations, and the total distance between facilities visited. We assessed temporal changes with linear regression and stratified results by location. Of 2,878 patients, 65% were from Cape Town. In Cape Town, 29% visited a TB hospital; elsewhere, 68% visited a TB hospital. We found that hospitalizations and travel distances were shorter in Cape Town than in the surrounding areas.

South Africa has a high tuberculosis (TB) prevalence, complicated by multidrug resistance to rifampin and isoniazid (*1*). In 2018, multidrug-resistant (MDR) and rifampin-resistant (RR) TB accounted for 3.4% of new and 7.1% of previously treated cases in South Africa (*1*). These forms of TB require more complex and lengthy treatments than drug-susceptible TB. Before 2011, most patients with MDR/RR TB in South Africa were hospitalized in dedicated TB hospitals, which were considered better than other facilities for managing infection control, regimen complexities, and side effects. However, centralized care might have contributed to delayed initiation of second-line drugs for MDR/RR TB, high pretreatment death rates caused by limited bed capacity, and patient loss to follow-up because of long-term hospitalization of clinically stable patients (*2**,**3*).

A 2009 pilot program in Khayelitsha township, Cape Town (*4*), South Africa, demonstrated that community-based care improved case detection. It also reduced death, health system costs, and treatment delays (*3**,**5**–**11*). In 2011, the South African National Department of Health implemented a national policy to decentralize and deinstitutionalize MDR/RR TB care (*2*). In Western Cape, MDR/RR TB care decentralization enabled clinically stable patients to initiate second-line TB treatment at 1 of 406 local facilities offering TB care instead of the province’s 6 specialized TB hospitals (*12*). The policy also reduced the required duration of TB hospitalizations for patients who required hospitalization (*2*). Because of the reduced density of TB hospitals outside Cape Town, the potential policy effects are largest in rural areas. However, long distances between facilities and lack of resources and experienced providers pose challenges to implementation in rural areas.

Despite these demonstrated benefits of decentralization, data analyzing its effects on hospitalization rates, duration, and travel distance in Western Cape are scarce. The National Health Laboratory Service (NHLS) conducts and records most laboratory tests in South Africa. We used NHLS data to track where patients received care for RR TB in the year after their diagnoses. We identified temporal trends in patient contact with TB hospitals, estimated hospital stay duration and distance traveled between facilities during early implementation of the national decentralization policy in Western Cape. We compared these metrics between Cape Town and more rural Western Cape districts.

## Methods

### Data Source

We extracted records of TB laboratory tests conducted on clinical samples at the NHLS TB laboratory in Green Point, Cape Town, during January 1, 2012–July 31, 2015. These tests were used to diagnose and monitor TB cases in the Western Cape. Samples originated from patients at various facilities, including specialized TB hospitals, primary healthcare clinics, mobile clinics, regional hospitals, and district hospitals. The NHLS records data on patients receiving tests through the public healthcare system, which conducts 93% of all TB tests nationally (*13*). The study was approved by Stellenbosch University’s Health Research Ethics Committee (protocol no. N09/11/296) and Boston University’s Institutional Review Board (no. H-38441). Given the study’s retrospective nature, an informed consent waiver was granted.

During the study period, the Western Cape’s TB investigation policy required that facilities submit 2 clinical samples from each patient to the nearest NHLS laboratory (*14*). Usually, the first sample was tested with Xpert MTB/RIF (Cepheid, https://www.cepheid.com). If RR TB was detected, the second sample was sent to the Green Point laboratory for smear microscopy, culturing with the mycobacterial growth indicator tube (Becton, Dickinson, and Company, https://www.bd.com), and drug susceptibility testing (DST). Line probe assays (LPAs) conducted by using GenoType MTBDR*plus* (Hain Lifescience GmbH, https://www.hain-lifescience.de) confirmed the presence of *Mycobacterium tuberculosis* and genes for resistance to rifampin and other first-line drugs. Phenotypic DST was used to detect genes conferring second-line drug (SLD) resistance. Although samples from tertiary (non-TB) hospitals with their own culture laboratories are not included in this dataset, the laboratory in Green Point conducts most culture-based and LPA confirmatory testing for TB in the Western Cape; therefore, this dataset includes most patients with RR TB in this province (Appendix).

Each NHLS record represents a single laboratory test but lacks a unique patient identifier. Therefore, to track patients over time, we used a patient matching algorithm to link samples belonging to the same patient. This algorithm, previously applied to NHLS HIV data, estimates the probability that records belong to the same patient on the basis of name, birthdate, sex, and facility data (*15*; J. Bor, unpub. data, https://www.biorxiv.org/content/early/2018/11/02/450304) (Appendix).

### Definitions

We defined a patient with RR TB as someone who submitted >1 clinical sample with bacteriological confirmation of *M. tuberculosis* and rifampin resistance according to Xpert MTB/RIF, LPA, phenotypic DST, or a combination of these testing methods at the NHLS laboratory in Green Point. We defined the taken date as the date the sample was obtained from a patient. We considered the taken date of the first RR TB–positive sample to be the patient’s initial sample date and the diagnosis date (Appendix). We defined a visit as a unique day in which a patient submitted >1 laboratory sample. Time in care was defined as 1 year from the initial RR TB sample or until the most recent sample in the study timeframe, whichever was earlier.

### Study Population

**Figure 1:**
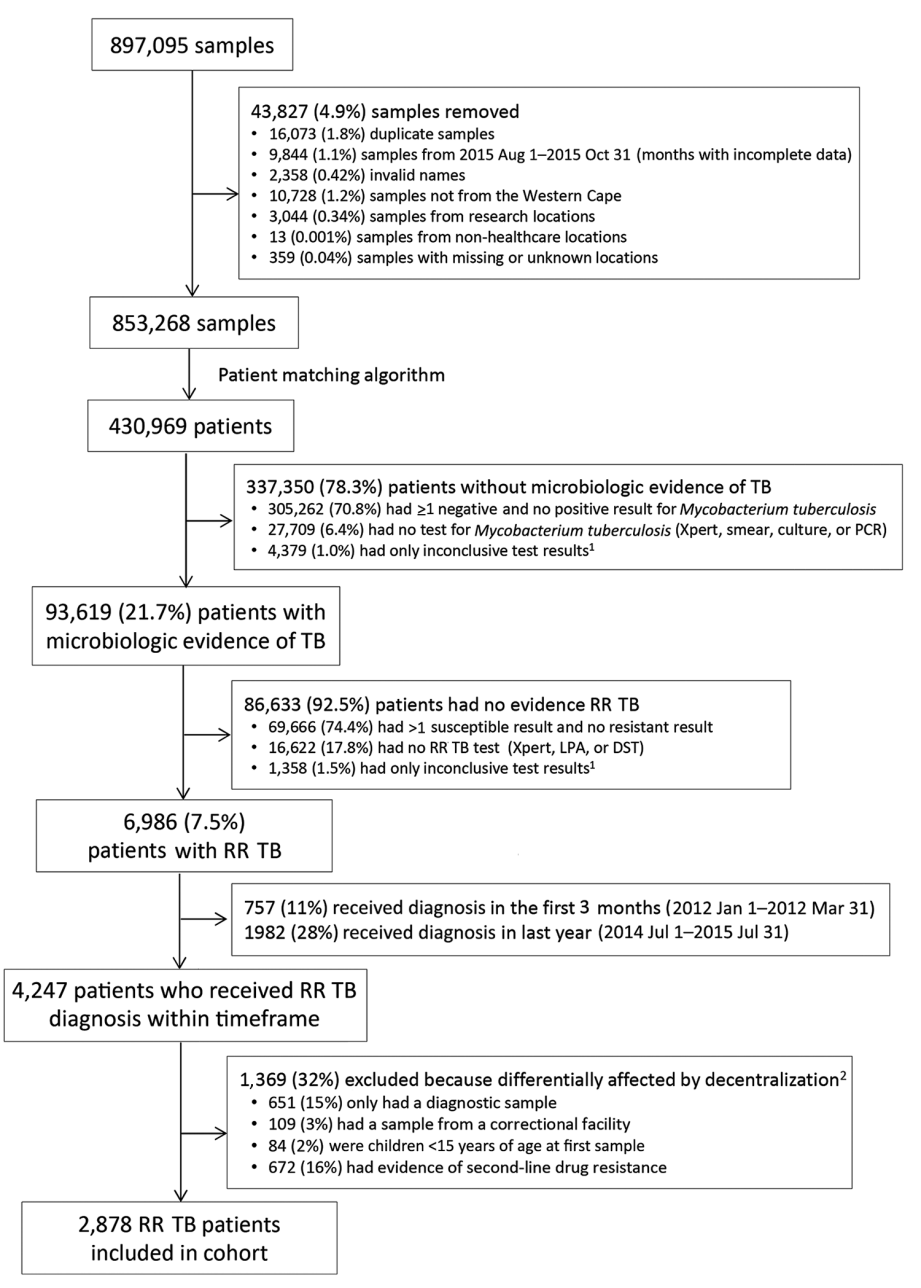
Flow diagram showing identification of adult patients with RR TB, Western Cape, South Africa, 2012–2014. Patients did not have second-line drug resistance and attended >2 clinic visits. The following test results were classed as inconclusive: inconclusive, error, unsuccessful, specimen container received empty, no result, lost viability, contaminated, specimen accidentally destroyed, insufficient specimen, or leaky specimen. The total number of patients excluded does not equal the sum of the individual categories because some patients belonged to multiple groups. RR, rifampin-resistant; TB, tuberculosis.

We analyzed each patient’s TB laboratory samples in the year after that patient’s initial RR TB sample was submitted to the NHLS during January 1, 2012–July 31, 2015. Using specific exclusion criteria (Figure 1), we excluded samples that were from locations outside Western Cape, collected for research purposes, submitted with invalid identifying data (e.g., names containing the words “control,” “staff,” “Ecoli”, etc.), or had facility codes that could not be linked to a physical location.

After linking samples to individual patients, we excluded patients whose initial RR TB sample was submitted after July 1, 2014, enabling us to study 12 months of follow-up for each patient. Some patients might have had less time in care if they died, moved out of the province, or were otherwise lost to follow-up, after which point these patients would no longer be included in the Western Cape public healthcare system. Because we could not correlate laboratory results with clinical records, we excluded patients whose initial RR TB sample was submitted during the first 3 months of the study (January 1–March 31, 2012) because this sample might not have been their diagnostic sample. We excluded patients who had no subsequent laboratory samples submitted to the NHLS because we assumed that those patients were less likely to have initiated treatment or stayed in care. Finally, we excluded patients who were less likely to have been affected by the decentralization policy: those in correctional facilities, those with documented SLD resistance, and children <15 years of age at diagnosis.

### Mapping Patient Movement

In the NHLS database, each sample is registered with a collecting facility code. We determined the facility name, type, and geocoordinates from NHLS and National Department of Health reference lists. We grouped facilities into 3 categories: specialized TB hospitals, non-TB hospitals (i.e., all other hospitals), and clinics (i.e., all other location types). We validated geocoordinates on Google Maps (https://maps.google.com); researchers and healthcare providers in South Africa resolved discrepancies. We combined facilities of the same type and geographic location into a single entity. We used the code associated with the samples from each patient to track patient movement between facilities.

### Decentralization Analysis

The national decentralization policy stated that clinically stable patients with no SLD resistance could initiate treatment at local hospitals and clinics designated as decentralized treatment initiation sites (*2*). According to this policy, although a small proportion of patients would still be hospitalized for clinical or psychosocial reasons, most patients with RR TB would be treated outside specialized TB hospitals. In addition, hospitalized patients would have shorter hospital stays (*2*).

We first summarized cohort characteristics regarding sex, age, TB type, type of facility submitting the initial RR TB sample, smear status, number of visits, and time in care. To assess decentralization implementation, we calculated the percentage of patients with >1 sample submitted from a TB hospital <1 year after diagnosis; we stratified these results by facility type (i.e., TB hospital, non-TB hospital, clinic). We calculated the percentage of patients who transitioned to care outside a TB hospital (i.e., patients who submitted samples from a non-TB hospital or clinic <3 months after their most recent sample from a TB hospital). For these patients, we estimated duration of TB hospitalization as the time between the date of the first sample submitted from the TB hospital to the midpoint between the most recent sample submitted from the TB hospital and the date of the first subsequent sample submitted from a clinic or non-TB hospital.

We then used simple linear regression to estimate temporal trends of all outcomes by quarter (i.e., 3-month period) of initial RR TB sample during April 2012–June 2014, for a total of 9 quarters. We ran 2 models for each outcome: 1 stratified by diagnosis location and 1 combined model with an interaction term to assess the differences in trend between locations. In addition, we used multivariable logistic regression to test the association between whether or not a patient submitted a sample from a TB hospital and quarter of initial RR TB sample adjusting for sex, age (15–34, 35–54, >55 years of age), TB type (pulmonary, extrapulmonary, both), smear status within 1 month of initial RR TB sample, and number of visits <1 year after diagnosis. For this analysis only, we excluded patients missing data on age, sex, or both.

One decentralization goal was to enable treatment closer to patients’ homes (*2*). We calculated the percentage of patients that had samples from ≥2 facilities, indicating movement between facilities. To estimate travel distance without home addresses, we calculated the total Euclidean distance between all facilities from which a patient submitted samples during the first year after diagnosis. For multiple visits, we counted distances multiple times. Because the number of visits could affect the total distance between facilities, we also determined each patient’s number of visits in the first year after diagnosis. We then controlled for the number of visits by calculating the median distance between facilities visited consecutively for each patient. We used linear regressions to assess temporal trends in these travel outcomes.

**Figure 2:**
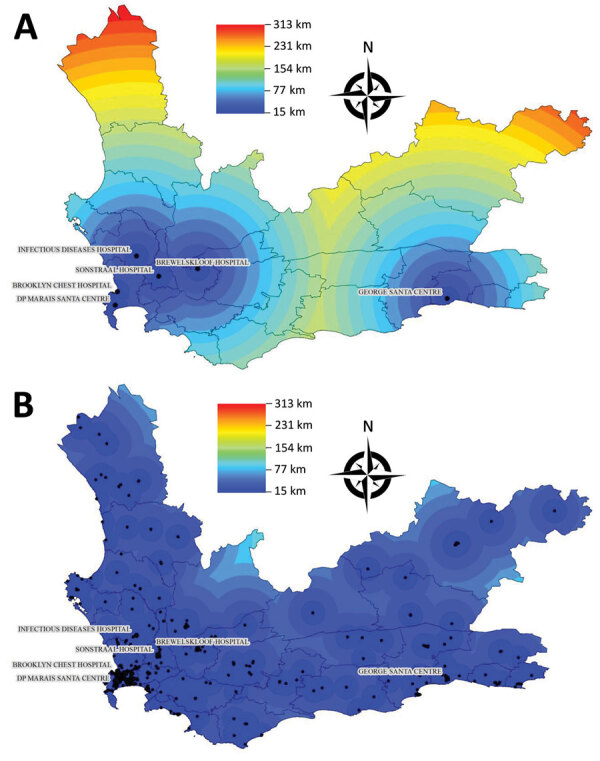
Distances to the nearest tuberculosis healthcare facility, Western Cape Province, South Africa. A) Distance to nearest specialized TB hospital. B) Distance to nearest facility of any type: TB hospital, clinic, or non-TB hospital that was visited by patients in this study during 2012–2015. TB, tuberculosis.

We stratified analyses by whether patients’ initial RR TB samples were from Cape Town or outside Cape Town (i.e., the rest of Western Cape) to identify differential implementation of decentralization. To demonstrate the potential travel benefit for patients receiving RR TB treatment in a clinic or local hospital compared with a specialized TB hospital, we mapped the distance to the nearest TB hospital from anywhere in the province and compared this distance to the distance to the nearest facility of any kind that submitted samples recorded in this study (Figure 2). We used R version 3.6.1 (*16*) for analyses and ArcMap version 10.6 (Environmental Systems Research Institute, Inc., https://desktop.arcgis.com) for mapping.

## Results

### Cohort Description

After excluding ineligible patients, we analyzed a cohort of 2,878 patients who received a diagnosis of RR TB during April 1, 2012–June 30, 2014 (Figure 1). The exclusions included 651 (15.3%) patients with only a diagnostic sample recorded (14.0% of patients in Cape Town and 17.7% outside Cape Town; Appendix Table 1). Of the 2,878 patients, 1,878 (65%) submitted their initial RR TB sample from Cape Town and 1,000 (35%) from outside Cape Town. The mean age was 36 years (SD ±12 years), and 57% were men. Most (93%) patients had RR TB detected from sputum or lung samples, suggesting pulmonary disease, and 49% had negative smear microscopy results when RR TB was detected (Table 1).

**Table 1:** Characteristics of patients with RR TB, Western Cape, South Africa, 2012–2014*

Characteristic†	Overall, n = 2,878	Cape Town, n = 1,878	Outside Cape Town n = 1,000	p value‡
Sex§				0.32
F	1,245 (43.4)	825 (44.1)	420 (42.0)	
M	1,626 (56.6)	1,047 (55.9)	579 (58.0)	
Age group, y¶				<0.01
15–34	1,420 (50.1)	978 (53.0)	442 (44.9)	
35–54	1,232 (43.5)	761 (41.2)	471 (47.8)	
>55	180 (6.4)	108 (5.8)	72 (7.3)	
Type of TB				0.72
Pulmonary only	2,685 (93.3)	1,747 (93.0)	938 (93.8)	
Extrapulmonary only	70 (2.4)	47 (2.5)	23 (2.3)	
Both	123 (4.3)	84 (4.5)	39 (3.9)	
Results of closest smear within 30 d of first RR TB–positive sample				0.93
Negative	1,396 (48.5)	913 (48.6)	483 (48.3)	
Scanty positive	310 (10.8)	202 (10.8)	108 (10.8)	
Positive +	262 (9.1)	178 (9.5)	84 (8.4)	
Positive ++	181 (6.3)	118 (6.3)	63 (6.3)	
Positive +++	499 (17.3)	321 (17.1)	178 (17.8)	
Unknown	230 (8.0)	146 (7.8)	84 (8.4)	
Setting of first RR TB–positive result				<0.01
TB hospital	103 (3.6)	43 (2.3)	60 (6.0)	
Clinic	2,361 (82.0)	1,554 (82.7)	807 (80.7)	
Non-TB hospital	414 (14.4)	281 (15.0)	133 (13.3)	
Median time in care** in first year after RR TB diagnosis, mos (IQR)	11 (5–12)	11 (5–12)	11 (6–12)	<0.01#
Median number of visits†† in the first year after RR TB diagnosis, (IQR)	9 (5–12)	8 (4–12)	10 (5–12)	<0.01#

*Patients without second-line drug resistance who attended >2 visits. RR, rifampin-resistant; TB, tuberculosis. †Data are no. (%), except where otherwise indicated. ‡p values determined by χ^2^ test of patients in Cape Town versus outside Cape Town. §A total of 7 patients were missing data on sex: 6 from Cape Town and 1 from outside of Cape Town. ¶A total of 46 patients were missing data on age: 31 from Cape Town and 15 from outside of Cape Town. #p values determined by Wilcoxon rank-sum test of patients in Cape Town versus outside Cape Town. **Time in care is defined as the time between the first and most recent RR TB–positive sample or 1 y from the first RR TB–positive sample, whichever was earlier. ††Defined as unique days in which a patient submitted >1 sample.

### Samples from Specialized TB Hospitals

In total, 2,361 (82%) patients submitted initial RR TB samples from clinics, 414 (14%) from non-TB hospitals, and 103 (4%) from TB hospitals. Although only 4% of patients submitted their initial RR TB sample from a TB hospital, 1,228 (43%) patients submitted >1 sample from a TB hospital <1 year after diagnosis. In particular, 894 (38%) patients who submitted their initial sample from a clinic and 231 (56%) who submitted their initial sample from a non-TB hospital submitted >1 additional sample from a TB hospital (Appendix Table 2). Patients in Cape Town were significantly less likely to submit a sample from a TB hospital than patients outside Cape Town (29% vs. 68%; p<0.01). Of the 545 patients from Cape Town who submitted a TB hospital sample, 317 (58%) transitioned to care outside of the TB hospital compared with 520 (76%) of the 683 patients outside Cape Town (p<0.01). We estimated that the median first TB hospital stay for those who transitioned to care outside of the TB hospital was 79 days (interquartile range [IQR] 50–118 days) in Cape Town and 108 days (IQR 72–144 days) outside Cape Town (Table 2).

**Table 2:** Magnitude and duration of hospitalization and movement of patients with RR TB, Western Cape, South Africa, 2012–2014*

Description	Overall, n = 2,878	Cape Town, n = 1,878	Outside Cape Town, n = 1,000	p value†
Hospitalization in TB hospital, no. (%) No. patients with >1 sample from a specialized TB hospital in the first year after RR TB diagnosis	1,228 (42.7)	545 (29.0)	683 (68.3)	<0.01
Moved to care outside TB hospital, no. (%) No. patients with a sample from a TB hospital who had a subsequent sample from a non-TB hospital <3 mo after the most recent sample in the TB hospital	837 (68.2)	317 (58.2)	520 (76.1)	<0.01
Median length of TB hospital stay, d (IQR) Median hospitalization period of patients who moved to care outside of a TB hospital in the first year after RR TB diagnosis	99 (61–136)	79 (50–118)	108 (72–144)	<0.01‡
Any movement, no. (%) No. patients who had samples from >2 different facilities in first year after RR TB diagnosis	1,765 (61.3)	1,012 (53.9)	753 (75.3)	<0.01
Median no. of visits (IQR) No. unique days with >1 laboratory sample in the first year after RR TB diagnosis	9 (5–12)	8 (4–12)	10 (5–12)	<0.01‡
Median total distance, km (IQR) Total Euclidian distance between all facilities visited by each patient in the first year after RR TB diagnosis	4.4 (0.0–41)	1.5 (0.0–20)	46.0 (0.2–122)	<0.01‡
Median distance between consecutive visits, km (IQR) Median distance between facilities visited consecutively by each patient in the first year after RR TB diagnosis	2.7 (0.0–19.8)	1.4 (0.0–12.2)	24.0 (0.2–64.8)	<0.01‡

*Patients without second-line resistance who attended >2 clinic visits. RR, rifampin-resistant; TB, tuberculosis. †χ^2^ p values of patients in Cape Town versus outside Cape Town. ‡p values determined by Wilcoxon rank-sum test.

**Figure 3:**
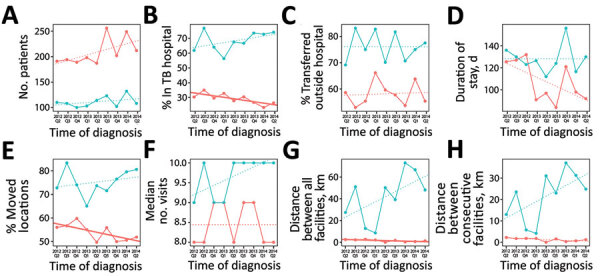
Linear time trends in magnitude and duration of movement for adult patients with RR TB, Western Cape, South Africa, 2012–2014. Patients did not have second-line drug resistance and attended >2 clinic visits. Linear regression trendlines are colored based on district of diagnosis (red indicates Cape Town; blue indicates other districts) and styled based on significance (solid line indicates p<0.05; dotted line indicates p>0.05). A) Number of patients diagnosed with RR TB. B) Percentage of patients who submitted a sample from a TB hospital <1 year after diagnosis. C) Percentage of patients who transitioned to care outside a TB hospital. D) Median duration of first stay in a TB hospital. E) Percentage of patients who transitioned to different facilities. F) Median number of visits in which patient submitted >1 sample. G) Median total Euclidean distance traveled between locations. H) Median Euclidean distance between consecutive visits. RR, rifampin-resistant; TB, tuberculosis.

In Cape Town, the percentage of patients who submitted a TB hospital sample in the first year on average decreased by 1 percentage point (95% CI 0.2%–1.7%; p = 0.02) per quarter, representing a 9 percentage point decrease during the study period; we observed no statistically significant trend outside Cape Town (Table 3). During the study period, the percentage of patients who transitioned to care outside of a TB hospital stayed constant in and outside Cape Town. In Cape Town, the estimated first TB hospital stay duration decreased by 3.6 days per quarter (95% CI –8.7 to 1.5 days; p = 0.14), for a total decrease of 32 days during the study. Outside Cape Town, the duration stayed constant (Table 3; Figure 3). Visual inspection of all trends indicated that linear trends were appropriate.

**Table 3:** Linear temporal trends in magnitude and duration of movement for adult patients with RR TB, Western Cape, South Africa, 2012–2014*

Description	Overall, n = 2,878			Cape Town, n = 1,878			Outside Cape Town, n = 1,000		Interaction p value†
	Slope (95% CI)	p value		Slope (95% CI)	p value		Slope (95% CI)	p value	
Hospitalization in TB hospital, no. (%) No. patients with >1 sample from a specialized TB hospital in the first year after RR TB diagnosis	−0.4 (−1.2 to 0.5)	0.33		−1.0 (−1.7 to −0.2)	0.02		1.1 (−0.9 to 3.1)	0.23	0.03
Moved to care outside TB hospital, no. (%) No. patients with a sample from a TB hospital who had a subsequent sample from a non-TB hospital <3 mo after the most recent sample in the TB hospital	0.2 (−0.9 to 1.3)	0.69		0.1 (−1.3 to 1.6)	0.84		−0.01 (−1.8 to 1.8)	0.99	0.89
Median length of TB hospital stay, d (IQR) Median hospitalization period of patients who moved to care outside of a TB hospital in the first year after RR TB diagnosis	−1.5 (−5.7 to 2.6)	0.42		−3.6 (−8.7 to 1.5)	0.14		−0.28 (−4.3 to 3.7)	0.87	0.24
Any movement, no. (%) No. patients who had samples from >2 different facilities in first year after RR TB diagnosis	−0.5 (−1.2 to 0.3)	0.19		−0.9 (−1.7 to −0.06)	0.04		0.5 (−1.2 to 2.3)	0.50	0.10
Median no. of visits (IQR) No. unique days with >1 laboratory sample in the first year after RR TB diagnosis	0.04 (−0.01 to 0.1)	0.12		0.0 (−0.2 to 0.2)	>0.99		0.1 (−0.01 to 0.2)	0.06	0.22
Median total distance, km (IQR) Total Euclidian distance between all facilities visited by each patient in the first year after RR TB diagnosis	−0.1 (−0.4 to 0.2)	0.43		−0.3 (−0.5 to −0.01)	0.04		4.7 (−1.3 to 10.6)	0.10	0.07
Median distance between consecutive visits, km (IQR) Median distance between facilities visited consecutively by each patient in the first year after RR TB diagnosis	−0.06 (−0.2 to 0.04)	0.21		−0.18 (−0.4 to −0.02)	0.07		2.5 (−0.5 to 5.6)	0.09	0.05

*Patients without second-line drug resistance who attended >2 visits. Estimates are change per quarter (i.e., 3 mos). RR, rifampin-resistant; TB, tuberculosis. †p value of interaction term between quarter of diagnosis and location.

We included 2,831 patients in the individual-level multivariable logistic regression analysis and adjusted for number of visits. In Cape Town, the odds of submitting a sample from a TB hospital decreased by 5% per quarter (p = 0.02); outside Cape Town, we found no statistically significant association. Outside Cape Town, increasing smear grade (i.e., scanty, +, ++, +++) was associated with increasing odds of submitting a sample from a TB hospital (Table 4).

**Table 4:** Multivariable logistic regression for factors associated with sample submitted from a TB hospital <1 y after diagnosis of RR TB, Western Cape, South Africa, 2012–2014*

Characteristic	Overall, n = 2,831			Cape Town, n = 1,846			Outside Cape Town, n = 985	
	OR (95% CI)	p value		OR (95% CI)	p value		OR (95% CI)	p value
Location								
Cape Town	Referent			Referent			Referent	
Other	5.7 (4.8–6.8)	<0.01		NA			NA	
Sex								
F	Referent			Referent			Referent	
M	1.2 (1.0–1.4)	0.03		1.2 (1.0–1.5)	0.07		1.2 (0.9–1.6)	0.27
Age, y								
15–34	Referent			Referent			Referent	
35–54	1.1 (0.9–1.3)	0.43		1.2 (0.9–1.5)	0.14		0.9 (0.7–1.3)	0.55
>55	0.8 (0.6–1.2)	0.24		1.0 (0.6–1.6)	0.95		0.6 (0.3–1.0)	0.05
Type of TB								
Pulmonary only	Referent			Referent			Referent	
Extrapulmonary only	1.0 (0.5–1.7)	0.98		0.8 (0.4–1.7)	0.63		1.3 (0.5–3.5)	0.53
Both	2.7 (1.8–4.2)	<0.01		3.7 (2.3–5.9)	<0.01		1.1 (0.5–2.4)	0.85
Results of most recent smear from <30 d of first RR TB–positive sample								
Negative	Referent			Referent			Referent	
Scanty positive	1.4 (1.0–1.8)	0.03		1.7 (1.2–2.4)	<0.01		1.0 (0.6–1.6)	>0.99
Positive +	1.5 (1.1–2.1)	<0.01		1.6 (1.1–2.2)	0.02		1.5 (0.9–2.8)	0.16
Positive ++	1.8 (1.3–2.5)	<0.01		1.5 (1.0–2.3)	0.06		3.0 (1.5–6.4)	<0.01
Positive +++	2.1 (1.7–2.7)	<0.01		1.9 (1.4–2.5)	<0.01		3.5 (2.2–5.8)	<0.01
Unknown	1.2 (0.9–1.7)	0.19		1.2 (0.8–1.9)	0.29		1.2 (0.7–2.1)	0.56
Quarter of RR TB diagnosis†	0.98 (0.95–1.02)	0.29		0.95 (0.92–0.99)	0.02		1.04 (0.98–1.10)	0.20

*Patients without second-line drug resistance who attended >2 visits. All analyses adjusted for no. of visits <1 y after diagnosis. Excludes 47 patients who are missing data on age, sex, or both. NA, not applicable; OR, odds ratio; RR, rifampin-resistant; TB, tuberculosis. †Estimated change per quarter (i.e., 3 mos).

### Distance Traveled

**Figure 4:**
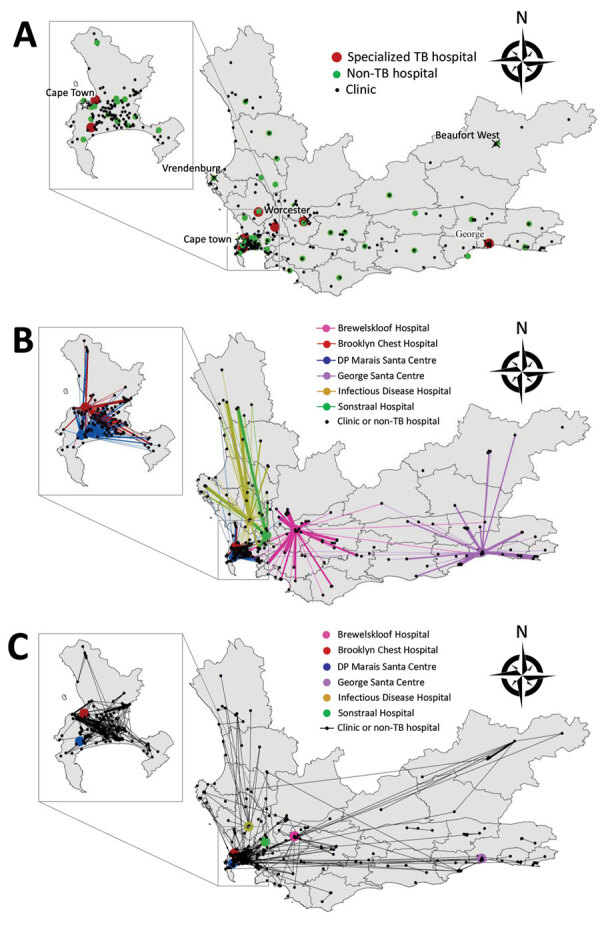
Healthcare facilities visited and movements between hospitals by patients in RR TB cohort, Western Cape Province, South Africa, 2012–2014. Inset maps show the Cape Town Metropole. A) All healthcare facilities visited <1 y after diagnosis. B) All movements made <1 y after diagnosis that involved TB hospitals. C) All movements made <1 y after diagnosis that did not involve a TB hospital. RR, rifampin-resistant; TB, tuberculosis.

In the first year after diagnosis, patients with RR TB had samples submitted from 315 different facilities: 268 clinics, 41 non-TB hospitals, and 6 TB hospitals (Appendix Table 3). Most patient movements between different facilities involved a TB hospital (Figure 4). A total of 1,765 (61%) patients submitted samples from >2 different facilities. Patients outside Cape Town were more likely to transition between facilities than those in Cape Town (75% vs. 54%; p<0.01) (Table 2). Overall, the median Euclidean distance traveled between facilities was 4.4 km (IQR 0–41 km). The median distance traveled was significantly shorter in Cape Town (1.5 km, IQR 0–20 km) than outside Cape Town (46 km, IQR 0.2–122 km; p<0.01). This disparity remained after controlling for the number of visits per patient (Table 2).

In Cape Town, the percentage of patients who transitioned between facilities decreased by 0.9 percentage points per quarter (95% CI 0.1%–1.7%; p = 0.04) and the total distance between all facilities visited decreased by 0.3 km per quarter (95% CI 0.01–0.5 km; p = 0.04). However, outside Cape Town, this distance increased by 4.7 km each quarter (95% CI –1.3 to 10.6 km; p = 0.10). We observed no statistically significant change in median number of visits. Trends in median distance between consecutive visits were consistent with total distance trends (Table 3; Figure 3). In Cape Town, the distances to the nearest TB hospital compared with the nearest clinic or non-TB hospital were similar. Outside Cape Town, clinics and non-TB hospitals were often much closer than the nearest TB hospital (Figure 2).

## Discussion

We used routinely collected laboratory data from Western Cape, South Africa to evaluate implementation of a national policy to decentralize MDR/RR TB care. Patients with RR TB in Cape Town facilities were less likely to have samples submitted from a TB hospital than patients outside Cape Town (29% vs. 68%, p<0.01), suggesting that persons in Cape Town were less likely to be hospitalized for RR TB. In addition, the percentage of patients who were likely hospitalized decreased significantly in Cape Town but not outside Cape Town. In Cape Town, the estimated average duration of TB hospitalization was nearly a month shorter and decreased over time compared with stays outside Cape Town, where duration remained constant.

These findings suggest that after the decentralization policy was implemented, more decentralization occurred in Cape Town than outside Cape Town. Loveday et al. (*9*) showed that treatment outcomes across decentralized sites in KwaZulu-Natal varied greatly and were highly influenced by health system performance. Health system factors such as long distances between facilities and limited provision of resources, training, and support from TB hospitals might have slowed decentralized care uptake in more rural areas. Furthermore, the large distances between patients in rural areas posed challenges to in-home medication administration. Additional outreach efforts such as mobile clinics have facilitated RR TB diagnosis. However, because mobile clinics might not be staffed in the same location each day, they are unable to administer SLDs, suggesting that broader access to new oral second-line TB drugs is needed in these settings (*17*).

Although the national policy change was introduced in 2011, Cape Town subdistricts had already begun decentralizing RR TB care after the success of the pilot program in Khayelitsha in 2009 (*2*,*4*). Our findings are consistent with previous work showing substantial challenges to healthcare access in rural areas of South Africa (*18**–**21*). The limited timeframe (2012–2015) of our study might have hindered our ability to detect slow changes in referral patterns outside Cape Town. However, Hill et al. (*18*) showed that in 2016, Cape Town patient travel patterns were still more consistent with a decentralized model than those elsewhere in the Western Cape.

In our study, patients outside Cape Town traveled 30 times further than patients in Cape Town (46 km vs. 1.5 km). Over the study period, travel distance decreased significantly for patients in Cape Town and increased for those outside Cape Town. This pattern of longer travel distances for healthcare in more rural areas of South Africa is well-documented (*18**–**20*). Although rural areas face more challenges to decentralization, the spread of local facilities throughout Western Cape indicates the potential for a reduction in travel distances for patients outside Cape Town (*12*). Shorter travel distances decrease treatment-related challenges for patients, enable local clinics to provide more patient support, and decrease risk for transmission during travel (*22*).

Although NHLS data are reliable for assessing aspects of TB and HIV care, its use introduces limitations to our study (*18*,*23**–**29*). These data lack information regarding treatment initiation, hospitalization, admission and discharge dates, and treatment outcomes. We therefore focused on where patients submitted samples and assumed repeat samples implied treatment prescription and monitoring (*29*,*30*). We also assumed that providing a sample at a TB hospital implied inpatient admission, which we believe is reasonable given that TB hospitals in the Western Cape only provide inpatient care (*12*). To focus on patients most likely to have started and continued RR TB treatment, we excluded patients without subsequent samples after the initial RR TB sample. However, this criterion might have excluded patients with extrapulmonary TB or those unable or unwilling to produce sputum samples. Furthermore, we could not account for patients who moved or transferred care to other provinces.

Without admission and discharge dates, our TB hospital stay duration estimate is a proxy for true hospital stay. In addition, without residential addresses, our distance traveled measure is a proxy for total travel distance. We also measured simple Euclidean distance between facilities, which might not reflect true traveling distance. Despite these limitations, the relative differences between Cape Town and outside Cape Town and the time trends should represent differences and trends in true hospital stays and travel distances.

Our study is also limited by its timeframe (2012–2015), which does not extend before the decentralization policy or to the present day, and by our inability to attribute causality between the decentralization policy and our estimated measures. Therefore, these results reflect patterns observed during early policy implementation and are a proof-of-concept that routinely collected laboratory data can be used to assess care patterns following policy implementation. However, other interventions, such as the introduction of GeneXpert, occurred in 2011 and 2012, which might also have affected TB diagnostic use and care. Our results might not be generalizable to all of South Africa because the Western Cape has more decentralized TB care units than other provinces (*12*), and Hill et al. (*18*) showed that in 2016 patients in Eastern Cape and KwaZulu-Natal had more centralized care patterns than patients in Western Cape.

The benefits of the decentralization of MDR/RR TB care have been documented in South Africa and elsewhere. In Khayelitsha, Cox et al. (*5**–**7*) found that decentralized care resulted in higher case detection, better outcomes, and lower costs. In KwaZulu-Natal, Loveday et al. (*9**–**11*) observed that decentralized sites had shorter time to treatment initiation and higher culture conversion rates; however, outcomes were poorer where decentralized services were not integrated into existing services. These studies concluded that regular monitoring and support were needed to optimize outcomes (*9**–**11*). Although Western Cape was the forerunner for implementing community-based MDR/RR TB care in South Africa, we have shown that locations outside Cape Town, and likely rural areas in general, need more support for implementing these policies (*12**,**18*). We have demonstrated a proof-of-concept that laboratory data can be used to assess policy implementation. As we work toward TB elimination, we must maximize our use of available, routinely collected data as a cost-effective, rapid method for evaluating policy implementation. Laboratory data can contribute to evidence-based expansion of policies to improve TB treatment and reduce incidence.

Appendix Further information on decentralized care for rifampin-resistant tuberculosis, South Africa.
