# Supplementary material for: Decentralized Care for Rifampin-Resistant Tuberculosis, Western Cape, South Africa
Source: Emerg Infect Dis. 2021 Mar;27(3):728–39. doi: 10.3201/eid2703.203204 (PMC7920662; doi:10.3201/eid2703.203204)
Supplement: Appendix — Further information on decentralized care for rifampin-resistant tuberculosis, South Africa. [file 20-3204-Techapp-s1.pdf]

Article DOI: https://doi.org/10.3201/eid2703.203204

Decentralized Care for Rifampin-Resistant Tuberculosis, Western Cape, South Africa

Appendix

Western Cape TB Testing Algorithm

During the study period, the local policy for tuberculosis (TB) investigation required that for every patient with suspected TB, 2 clinical samples (e.g., sputum, gastric washing or lavage, lymph node fine needle aspirate, pleural biopsy, cerebrospinal fluid) should be sent to the nearest National Health Laboratory Service (NHLS) location for testing with GeneXpert MTB/RIF (Cepheid, https://www.cepheid.com) (*1*). When rifampin-sensitive *Mycobacterium tuberculosis* was detected, the local laboratory would use the second sample for smear microscopy for monitoring purposes. However, if the sample was rifampin-resistant, the local laboratory would send the second sample to the NHLS TB laboratory in Green Point for smear microscopy, culturing with mycobacterial growth indicator tubes (Becton, Dickinson, and Company, https://www.bd.com), and drug susceptibility testing (DST) by GenoType MTBDR*plus* and GenoType MTBDR*sl* (Hain Lifescience GmbH, https://www.hain-lifescience.de). GenoType MTBDR*plus* (a line probe assay) was used to identify mutations conferring resistance to rifampin and isoniazid and GenoType MTBDR*sl* was used only on cultured isolates to identify mutations conferring resistance to fluoroquinolones and second-line drugs. However, GeneXpert MTB/RIF was not routinely used as the initial diagnostic test in patients with a history of TB in the previous 2–5 years; instead, samples from patients with recent TB history were sent to the NHLS for smear, culture, and DST using GenoType MTBDR*plus* and GenoType MTBDR*sl*. Only GenoType MTBDR*plus* and not Xpert was used to identify rifampin resistance.

Identifying Individual Patients in the NHLS Data

Each record within the NHLS database represents a single laboratory test on a clinical sample (sputum and other samples). Because patients can receive multiple different baseline tests

to identify TB and rifampin-resistant TB (RR TB) and are monitored at regular, ideally monthly, intervals during treatment (through submission of samples for smear microscopy and culturing), each patient can be associated with multiple records in the NHLS database. The NHLS database does not include a unique patient identifier; therefore, we used a patient matching algorithm to link all test results belonging to an individual patient.

We applied a method that we had previously developed and tested for the NHLS HIV database (*2*; J. Bor, unpub. data, https://www.biorxiv.org/content/10.1101/450304v1). Our method uses the first name, last name, birthdate, sex, and facility recorded for each sample in the NHLS database and applies probabilities that similar inputs are actually the same person. We combined the Fellegi-Sunter method of probabilistic record linkage with graph(network)-based concepts to assess the possibility that results belonged to unique patients. The Fellegi-Sunter approach assigns scores for pairwise comparisons of laboratory results across the identifying characteristics vector, with greater weight assigned to matches on rarer response options, such as rare names, that are unlikely to occur by chance. The Jaro-Winkler string comparison function assesses name similarity and was integrated into the Fellegi-Sunter approach.

Because probabilistic linkage can lead to overmatching in large datasets, graph concepts guide the linkage, improving accuracy and the scalability of the approach to the NHLS database. In the graphical approach, each set of identifiable information is a node and the edges connecting these nodes are assigned weights according to the similarity scores transformed to a 0–1 scale. We defined a threshold of similarity to identify which samples belong the same patient. To choose a threshold, we used a manually matched subset of patients to calculate the sensitivity (the proportion of true matches in the manually matched set that are identified as matches by the algorithm’s ID) and positive predictive value (the proportion of matches identified by the algorithm’s ID that are true matches based on the manually matched set) at each threshold (Appendix Figure).

For our dataset, we chose a threshold of 0.8 because this threshold resulted in the highest proportion of correct results on manual matching and also optimized the positive predictive value and sensitivity (Appendix Figure 1). We carried out sensitivity analyses across multiple thresholds comparing case counts, hospitalization percentages, movement percentages, and

trends in hospitalization and movement over time. We found no substantive change in our results (Appendix Table 4).

Definition of Dates

We defined the taken date of a sample as the date it was obtained from a person in a health facility and the registered date as the date the sample was received in the laboratory. If the taken date was not available (as in 1% of samples), or was >60 days before the registered date (as in 0.05% of samples), we imputed the taken date from the registered date by subtracting one day as this was the median difference between those dates for samples that had both. The taken date of the first RR TB–positive sample from each person was considered the date of the initial RR TB sample and the date of RR TB diagnosis.

References

1. National Department of Health. National tuberculosis management guidelines 2014. Pretoria, South Africa: Fishwicks PTA; 2014. p. 28.

2. Fox MP, Bor J, Brennan AT, MacLeod WB, Maskew M, Stevens WS, et al. Estimating retention in HIV care accounting for patient transfers: a national laboratory cohort study in South Africa. [Erratum in: PLoS Med. 2018;15:e1002643]. PLoS Med. 2018;15:e1002589. PubMed https://doi.org/10.1371/journal.pmed.1002589

Appendix Table 1. Distribution of patients with rifampin-resistant tuberculosis who were excluded from study, Western Cape, South Africa

Characteristic, no. (%)	Total n = 4,247	Cape Town n = 2,756	Outside Cape Town n = 1,491	p value*
Provided diagnostic sample only	651 (15.3)	386 (14.0)	265 (17.8)	<0.01
Sample sent from correctional facility	109 (2.6)	57 (2.1)	52 (3.5)	<0.01
Age <15 y†	84 (2.0)	34 (1.2)	50 (3.6)	<0.01
Any second-line drug resistance	672 (15.8)	496 (18.0)	176 (11.8)	<0.01
Total excluded‡	1,369 (32.2)	878 (31.9)	491 (32.9)	0.48

*p values determined by χ^2 test of patients in Cape Town versus other districts.
†At time of first sample.
‡The total excluded does not equal the sum of the individual categories because some patients belonged to multiple groups.

Appendix Table 2. Hospitalization percentages of adult patients with rifampin-resistant TB, Western Cape, South Africa, 2012–2014*

Setting of first rifampin-resistant TB–positive sample	Patients submitting ≥1 samples from a TB hospital, no. (%)		
	Overall	Cape Town	Outside Cape Town
TB hospital	103 (100.0)	43 (100.0)	60 (100.0)
Clinic	894 (37.9)	366 (23.6)	528 (65.4)
Non-TB hospital	231 (55.8)	136 (48.4)	95 (71.4)
Total	1,228 (42.7)	545 (29.0)	683 (68.3)

*Patients with no second-line drug resistance who attended ≥2 visits. TB, tuberculosis.

Appendix Table 3. Facilities visited by adult patients with rifampin-resistant tuberculosis, Western Cape, South Africa, 2012–2014*

District, subdistrict	TB hospitals	Non-TB hospitals	Clinics	Samples	Patients†
City of Cape Town					
Cape Town Eastern	0	2	15	2,397	324
Cape Town Northern	0	0	11	1,207	145
Cape Town Southern	1	2	20	2,031	402
Cape Town Western	1	4	16	2,320	456
Khayelitsha	0	1	8	2,585	361
Klipfontein	0	1	12	1,963	299
Mitchells Plain	0	2	12	2,023	327
Tygerberg	0	3	16	2,231	319
Subtotal	2	15	110	16,757	2,633
Cape Winelands					
Breede Valley	1	1	12	1,882	264
Drakenstein	1	1	17	1,056	179
Langeberg	0	2	7	214	36
Stellenbosch	0	1	10	492	65
Witzenberg	0	1	9	440	72
Subtotal	2	6	55	4,084	616
Central Karoo					
Beaufort West	0	1	7	300	41
Laingsburg	0	1	1	14	4
Prince Albert	0	0	1	37	5
Subtotal	0	2	9	351	50
Eden					
Bitou	0	0	5	219	36
George	1	2	11	1,563	258
Hessequa	0	1	4	78	15
Kannaland	0	1	3	52	12
Knysna	0	1	5	203	31
Mossel Bay	0	1	7	282	53
Oudtshoorn	0	1	6	234	41
Subtotal	1	7	41	2,631	446
Overberg					
Cape Agulhas	0	1	2	70	13
Overstrand	0	1	6	216	32
Swellendam	0	1	5	75	14
Theewaterskloof	0	1	7	370	63
Subtotal	0	4	20	731	122
West Coast					
Bergrivier	0	2	3	85	14
Cederberg	0	2	5	192	35
Matzikama	0	1	9	568	83
Saldanha Bay	0	1	9	352	50
Swartland	1	1	7	443	140
Subtotal	1	7	33	1,640	322
Total	6	41	268	26,194	4,189

*Patients with no second-line drug resistance who attended ≥2 visits.
†Total no. of patients from each subdistrict who provided samples; some patients are counted twice.

Appendix Table 4. Different patient matching algorithm thresholds for patients with RR TB, Western Cape, South Africa, 2012–2014*

Characteristic	Chosen threshold (0.8)	Tested ranges† Full range (0–0.99)	Narrow range (0.5–0.95)	Narrower range (0.7–0.9)
Case counts				
All patients	430,969	423,013–438,459	428,786–433,766	430,268–432,627
Patients with TB	93,619	92,291–95,436	93,208–94,258	93,483–93,973
Patients with RR TB	6,986	6,825–7,348	6,909–7,094	6,964–7,041
Study cohort	2,878	2,844–2,943	2,858–2,899	2,874–2,894
Location and setting of first RR TB–positive sample				
Location				
Cape Town	1,878	1,858–1,913	1,865–1,893	1,874–1,886
Other districts	1,000	986–1,030	993–1,006	1,000–1,008
Setting, %				
Clinic	82.0	82.1–80.8	82.1–81.7	82.0–81.8
Non-TB hospitals	14.4	14.6–14.4	14.5–14.6	14.4–14.5
TB hospitals	3.6	3.3–4.8	3.5–3.7	3.6–3.7
Hospitalization and movement proportions‡				
Sample from a TB hospital, %				
Overall	42.7	43.0–39.6	42.9–41.7	42.8–42.2
Cape Town	29.0	29.1–27.2	29.1–28.4	29.1–28.6
Other districts	68.3	69.1–62.4	68.9–66.8	68.4–67.6
Any movement, %				
Overall	61.3	62.7–56.7	62.0–60.3	61.5–60.7
Cape Town	53.9	54.9–50.7	54.3–53.1	54.1–53.5
Other districts	75.3	77.3–67.9	76.5–73.9	75.5–74.3
Median total distance between locations, km				
Overall	4.4	5.3–2.6	4.8–3.9	4.5–4.1
Cape Town	1.5	1.8–0.9	1.6–1.4	1.6–1.5
Other districts	46.1	52.2–13.6	48.1–41.6	46.8–43.9
Hospitalization and movement trends				
Sample from a TB hospital, slope (p)				
Cape Town	−1.0 (0.02)	−1.1 (0.01) to −1.0 (0.04)	−1.0 (0.02) to −1.0 (0.03)	−1.0 (0.02) to −1.0 (0.01)
Other districts	1.1 (0.23)	1.1 (0.25)−0.5 (0.48)	1.2 (0.19)−0.9 (0.27)	1.1 (0.23)−1.1 (0.22)
Any movement, slope (p)				
Cape Town	−0.9 (0.04)	−0.8 (0.05) to −0.8 (0.14)	−0.9 (0.04) to −0.9 (0.05)	−0.9 (0.03) to −0.9 (0.04)
Other districts	0.5 (0.50)	0.4 (0.57) to −0.2 (0.78)	0.7 (0.33)−0.4 (0.58)	0.5 (0.48)−0.5 (0.56)
Total km between locations, slope (p)				
Cape Town	−0.3 (0.04)	−0.2 (<0.01) to −0.2 (0.02)	−0.2 (0.01) to −0.3 (0.03)	−0.2 (0.01) to −0.2 (0.05)
Other districts	4.7 (0.10)	4.1 (0.15)−2.7 (0.18)	4.6 (0.11)−4.3 (0.18)	4.5 (0.13)−4.8 (0.09)

*Patients with no second-line drug resistance. RR TB, rifampin-resistant tuberculosis.
†The ranges throughout the table correspond to the lower matching threshold and the higher matching threshold; the lower threshold does not necessarily correspond to the lower value.
‡Movement between care facilities.

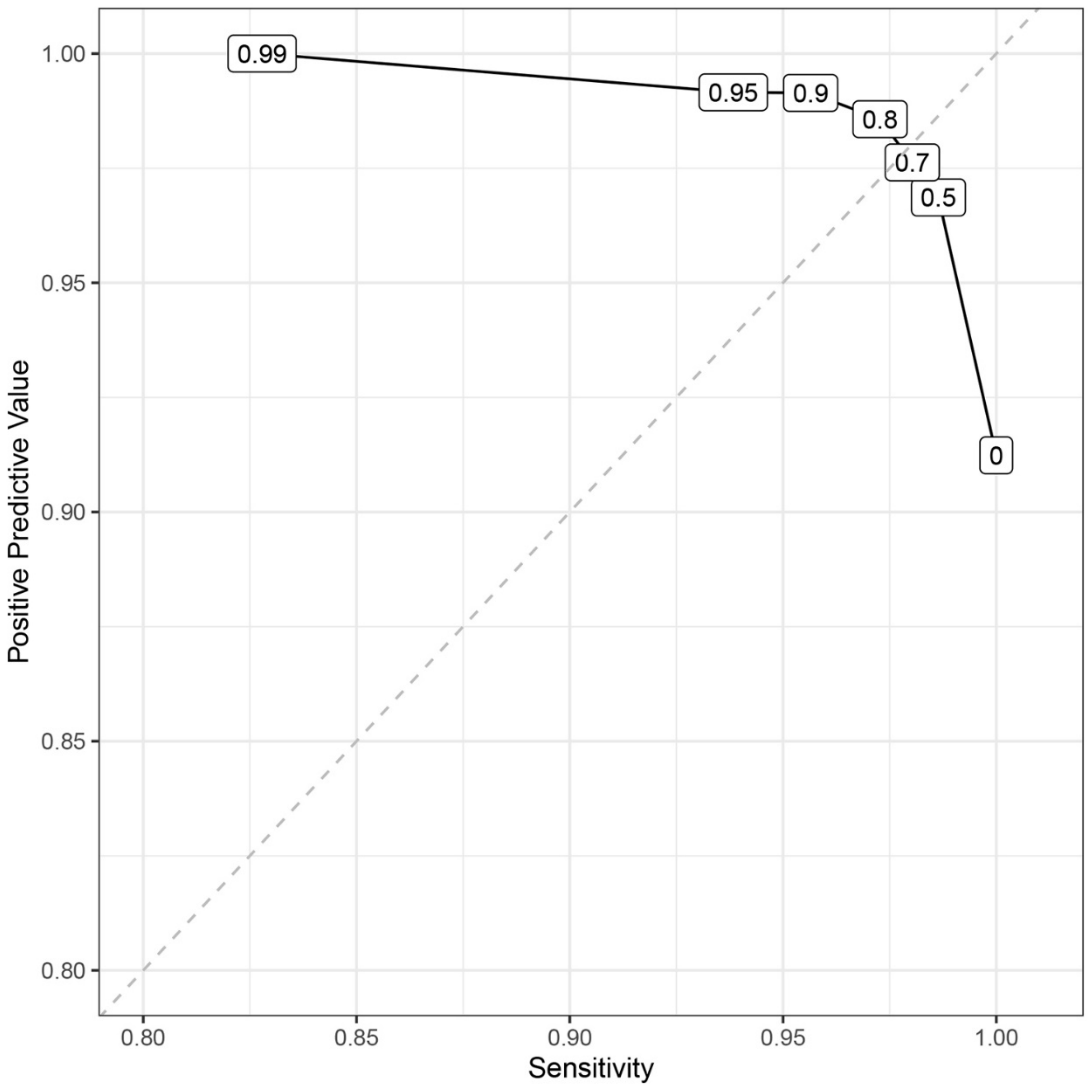

Appendix Figure. Receiver operating curve of different thresholds for the patient matching algorithm for patients with rifampin-resistant tuberculosis, Western Cape, South Africa, 2012–2014.
